# Hypoxia-NOTCH1-SOX2 signaling is important for maintaining cancer stem cells in ovarian cancer

**DOI:** 10.18632/oncotarget.10954

**Published:** 2016-07-30

**Authors:** Eun Jin Seo, Dae Kyoung Kim, Il Ho Jang, Eun Jung Choi, Sang Hun Shin, Su In Lee, Sang-Mo Kwon, Ki-Hyung Kim, Dong-Soo Suh, Jae Ho Kim

**Affiliations:** ^1^ Department of Physiology, School of Medicine, Pusan National University, Yangsan 50612, Gyeongsangnam-do, Republic of Korea; ^2^ Department of Obstetrics and Gynecology, School of Medicine, Pusan National University, Yangsan 50612, Gyeongsangnam-do, Republic of Korea; ^3^ Research Institute of Convergence Biomedical Science and Technology, Pusan National University Yangsan Hospital, Yangsan 50612, Gyeongsangnam-do, Republic of Korea

**Keywords:** cancer stem cells, ovarian cancer, hypoxia, NOTCH1, SOX2

## Abstract

Hypoxia and NOTCH signaling have been reported to be associated with the self-renewal and drug resistance of cancer stem cells (CSCs). However, the molecular mechanisms by which hypoxia and NOTCH signaling stimulate the self-renewal and drug resistance of ovarian CSCs are poorly understood. In the present study, we identified SOX2 as a key transcription factor for CSC-like characteristics in the downstream of hypoxia-induced NOTCH signaling in epithelial ovarian cancer cells. Hypoxic treatment or overexpression of intracellular domain of NOTCH1 (NICD1) in ovarian cancer cells increased sphere formation, drug resistance, and expression of CSC-associated genes such as SOX2, ALDH, and ABC transporters. Hypoxic treatment increased the expression of NICD1, and hypoxic treatment or NICD1 overexpression increased SOX2 promoter activity, which was inhibited by deletion of HIF-1 or CSL binding sites. Furthermore, DAPT treatment decreased the effect of hypoxic treatment, and SOX2 knockdown decreased the effect of hypoxic treatment and NICD overexpression on sphere formation and drug resistance in established ovarian cancer cell lines and primary ovarian cancer cells. These results suggest that hypoxia-NOTCH1-SOX2 signaling axis is important for activation of ovarian CSCs, which may provide a novel opportunity for developing therapeutics to eradicate CSCs in ovarian cancer patients.

## INTRODUCTION

Ovarian cancer is the leading cause of death among cancer-related diseases in women [1]. Though ovarian cancer is initially chemoresponsive, the majority of patients experience relapses with chemoresistance within 5 years, which renders ovarian cancer as a cancer stem cell (CSC)-related disease [2]. CSCs have been shown to be enriched in sphere cultures, which are often more resistant to chemotherapeutic reagents [3]. In ovarian cancer, CSC-enriched spheres are considered to disseminate cancers in the peritoneal cavity and cause recurrence with drug resistance [4]. It has been shown that the drug resistance property of CSCs is conferred by increased expression of multidrug resistance proteins, such as ABCG2 [5]. In addition, accumulating evidences suggests aldehyde dehydrogenase 1 (ALDH1) as a marker of ovarian CSCs [6]. Ovarian cancer cells with high ALDH1 expression displayed CSC-like properties, such as anchorage-independent growth and enhanced drug resistance [7]. As the main source of relapse in ovarian cancer has been attributed to CSCs, the necessity for development of a new therapeutics to eradicate CSCs has been increasing.

Hypoxic microenvironments have been identified in many solid tumors and implicated in development of resistance to chemotherapeutic agents [8]. Hypoxia signaling stimulates stemness-related pathways including OCT4, SOX2, NOTCH, and ABC transporters, which are responsible for multiple drug resistance [9]. Hypoxia-inducible factor-1a (HIF-1α), which is induced by hypoxia, confers protection of cancer cells against chemotherapeutic drugs-induced cell death [10]. After binding to the cognate enhancer sequence, HIF-1α activates the transcription of various genes required for tumorigenesis and metastasis [11]. In ovarian cancer, hypoxic microenvironment is known to increase CSC characteristics including sphere formation, the higher proliferation, the higher infiltration, and expression of OCT4 and SOX2 [12]. In spite of importance of hypoxic microenvironment in stimulating CSC-like characteristics, the molecular mechanisms by which hypoxia promotes generation of CSCs in ovarian cancer are still elusive.

NOTCH signaling is involved in various cellular processes during embryo development and stem cell maintenance in adult tissue [13, 14]. NOTCH signaling is activated upon ligand binding and release of NOTCH1 intracellular domain (NICD1), which translocates to the nucleus and binds to a DNA-binding transcription factor CSL [15]. Aberrant activation of NOTCH signaling has been identified in multiple cancers including leukemia, breast, brain, colorectal, and ovarian cancer [16]. Recent studies of NOTCH signaling in ovarian cancer have found a critical role of NOTCH1 in proliferation and metastasis of ovarian cancer cells. Overexpression of NICD1 enhanced proliferation of ovarian carcinoma cells, and NICD1 expression was identified in 76% of primary ovarian tumors [17, 18]. Association of activation of NOTCH1 signaling with chemoresistance and metastasis of ovarian carcinoma has been reported [19, 20]. In addition, increased expression of NOTCH1 was identified as an independent prognostic factor for poor survival of epithelial ovarian cancer patients [21]. However, NOTCH downstream effectors in ovarian cancer, particularly in CSCs, have not been clearly identified.

SOX2 is a transcription factor with characteristics of regulating self-renewal and differentiation of embryonic stem cells and reprogramming of somatic cells to pluripotent stem cells [22, 23]. Accumulating data have shown elevated expression of SOX2 in several types of CSCs, including breast, gastric, lung, and ovarian cancer [24]. In human ovarian carcinoma, SOX2 expression showed correlation with stem cell state [25]. SOX2 expression was increased in tumor spheres, and overexpression of SOX2 stimulated CSC properties including stemness marker expression, sphere formation, *in vivo* tumorigenicity, and resistance to apoptosis. However, the regulation mechanism of SOX2 expression in the ovarian CSC population has not been understood.

In the current study, we explored the roles of hypoxia, NOTCH1, and SOX2 in the sphere-forming ability, drug resistance, and CSC marker expression of CSC-like cells isolated from ovarian cancer cell lines and primary ovarian cancer cells. We demonstrated that hypoxia-NOTCH1-SOX2 signaling axis activates the acquisition of CSC-like characteristics in ovarian cancer cells.

## RESULTS

### SOX2 expression is increased in sphere-forming ovarian CSCs

**Figure 1:**
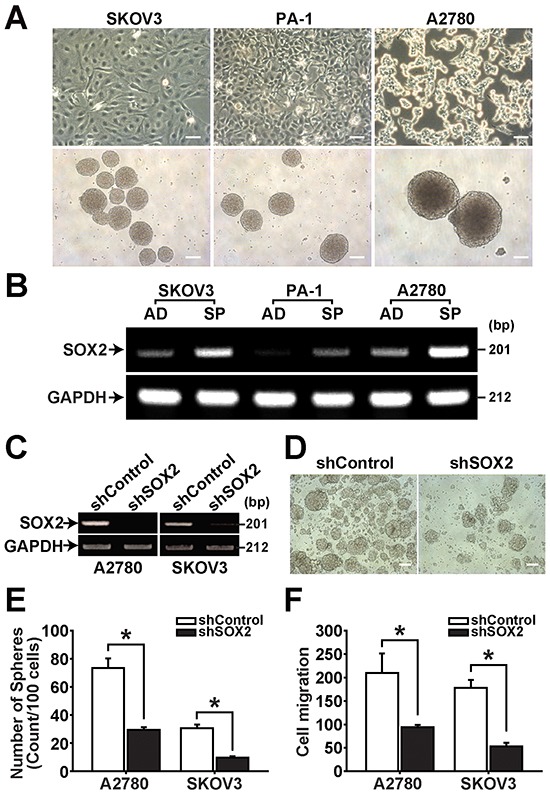
SOX2 expression is increased in spheres of ovarian cancer cells **A.** Spheres were generated from confluent culture of adherent SKOV3, PA-1, and A2780 cells (upper panels) and maintained in suspension culture (lower panels). Spheres were photographed using an inverted microscope on day 7 after individual sphere cells were seeded into low attachment 6-well plates. Scale bar = 100 μm. **B.** RT-PCR results of adherent (AD) and sphere cells (SP) with indicated probes are shown. **C.** RT-PCR results of A2780-SP and SKOV3-SP cells with or without SOX2 knockdown are shown with indicated probes. **D.** Representative images of spheres generated from A2780-SP cells with or without SOX2 knockdown are shown. Scale bar = 100 μm. **E.** Numbers of spheres generated from A2780-SP or SKOV3-SP cells with or without SOX2 knockdown are shown. Data indicate mean ± SD (n=4). *, P<0.05. **F.** Migration of A2780-SP or SKOV3-SP cells with or without SOX2 knockdown was measured using the Boyden chamber assay. Data indicate mean ± SD (n=4). *, P<0.05.

CSCs have been suggested to possess anchorage-independent growth and sphere-forming abilities in a serum-deprived suspension culture [4, 26]. We have recently reported isolation of sphere-forming CSCs from several epithelial ovarian cancer cell lines and primary ovarian cancer cells [27, 28]. In the present study, we isolated sphere-forming cells from three ovarian cancer cell lines, SKOV3, PA-1, and A2780 cells, by culturing cells in CSC culture medium (Figure 1A). In measurement of SOX2 expression by RT-PCR and immunocytochemistry, spheres (SP) derived from A2780, SKOV3, PA-1 showed the increase of SOX2 expression compared with their adherent cells (AD) (Figure 1B and Supplementary Figure S1). Knockdown of SOX2 expression using shRNA decreased sphere-forming ability of A2780 and SKOV3 cells along with reduced cell migration (Figure 1C–1F). On the contrary, overexpression of SOX2 enhanced sphere formation in A2780 and SKOV3 cells (Supplementary Figure S2). These results suggest that SOX2 stimulates sphere forming activity in ovarian cancer cells.

### SOX2 expression is involved in chemoresistance of CSCs through expression of ABC transporters

**Figure 2:**
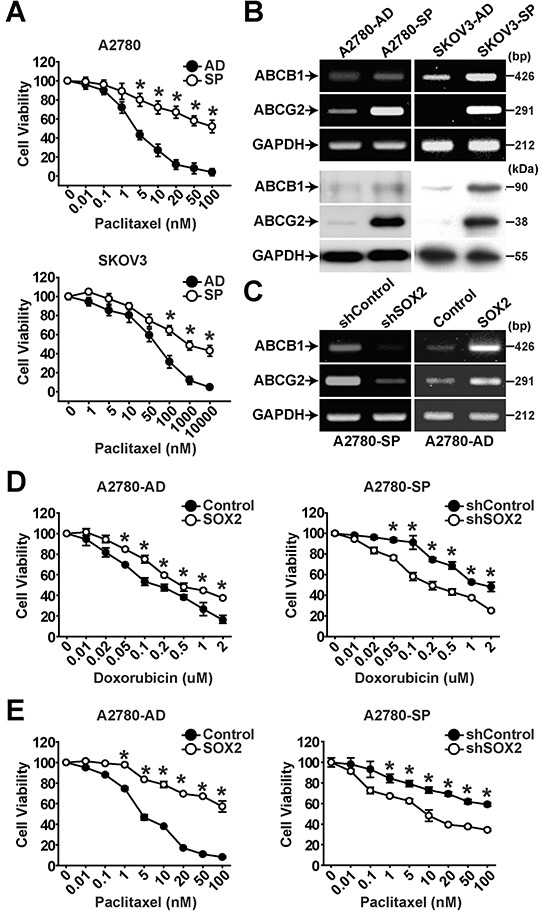
SOX2 expression is important for maintaining chemoresistance in ovarian cancer cells **A.** Viability of adherent cells (AD) or sphere cells (SP) of A2780 (upper panel) or SKOV3 (lower panel) ovarian cancer cells in the presence of increasing concentrations of paclitaxel was determined by MTT assay. The percentage of viable cells is shown after normalization to no treatment control. Data indicate mean ± SD (n=4). *, P<0.05. **B.** Results of RT-PCR analysis (upper panels) and Western blotting analysis (lower panels) with indicated probes in adherent and sphere cells of A2780 or SKOV3 ovarian cancer cells are shown. **C.** Results of RT-PCR analysis with indicated probed in A2780 sphere cells with or without SOX2 knockdown (left panels) or A2780 adherent cells with or without SOX2 overexpression (right panels) are shown. **D, E.** Viability of A2780 sphere cells with or without SOX2 knockdown or A2780 adherent cells with or without SOX2 overexpression in the presence of doxorubicin **(D)** or paclitaxel **(E)** was determined by MTT assay. The percentage of viable cells is shown after normalization to no treatment control. Data indicate mean ± SD (n=4). *, P<0.05.

Resistance of CSCs derived from many cancers to a variety of chemotherapeutic agents has been previously demonstrated [29]. In evaluation of drug resistance of adherent cells and spheres of A2780 or SKOV3 cells, spheres showed the higher resistance to paclitaxel compared with their adherent cells (Figure 2A). In assessment of expression of drug transporters by RT-PCR and Western blotting, spheres showed the higher expression of ABCB1 and ABCG2 than adherent cells (Figure 2B). Paclitaxel treatment time-dependently increased the protein levels of ABCB1 and ABCG2 in adherent cells and spheres of A2780 cells (Supplementary Figure S3). Knockdown of SOX2 expression in A2780 spheres (A2780-SP) resulted in significantly decreased expression of ABCB1 and ABCG2, whereas overexpression of SOX2 in A2780 adherent cells (A2780-AD) increased expression of ABCB1 and ABCG2 (Figure 2C and Supplementary Figure S4). In addition, knockdown of SOX2 expression in spheres resulted in significantly decreased resistance to doxorubicin or paclitaxel whereas overexpression of SOX2 in A2780-AD increased resistance to doxorubicin or paclitaxel (Figure 2D and 2E). These results suggest that SOX2 increases drug resistance through activating the expression of ABCB1 and ABCG2 in ovarian cancer cells.

### Hypoxia promotes acquisition of CSC-like characteristics in ovarian cancer

**Figure 3:**
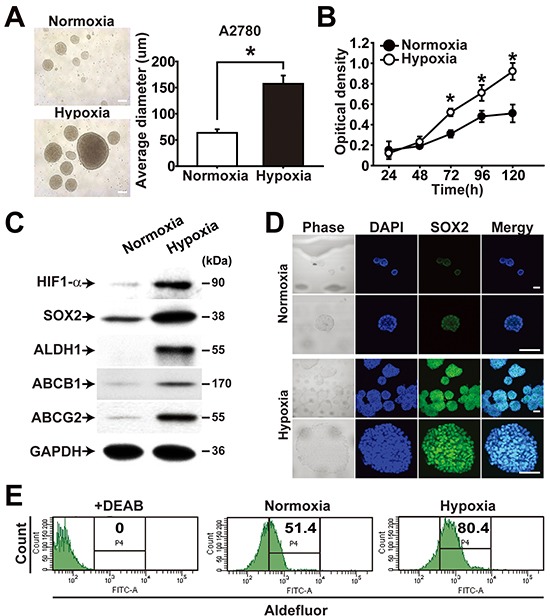
Hypoxia enhances CSC properties of ovarian cancer cells **A.** Representative images (left panel) and average diameter (right panel) of spheres from A2780 ovarian cancer cells cultivated under normoxia (20% O_2_) or hypoxia (1% O_2_) are shown. Scale bar = 100 μm. **B.** Growth curves of A2780 sphere cells cultivated under normoxia or hypoxia are shown. Data indicate mean ± SD (n=4). *, P<0.05. **C.** Western blotting results of A2780 spheres cultivated under normoxia or hypoxia with indicated probes are shown. **D.** Representative images from immunocytochemistry of A2780 spheres cultivated under normoxia or hypoxia are shown. Confocal images of A2780 sphere cells after immunolabeling with anti-SOX2 antibody are shown. Nuclei were stained with DAPI. Scale bar = 100 μm. **E.** Flow cytometry analysis results of A2780 sphere cells cultivated under normoxia or hypoxia for 120 h are shown. ALDH activity was measured after incubation of cells with Aldefluor. DEAB treatment in cells under normoxia is a negative control.

Hypoxic microenvironment was shown to stimulate stem-like properties in ovarian cancer cells [12]. When A2780-SP cells were subjected to hypoxic culture (1% O_2_), sphere generation and cell proliferation were augmented compared with incubation in normoxic condition (Figure 3A and 3B). Spheres cultured in a hypoxic chamber showed elevated expression of HIF-1α, SOX2, ALDH1, ABCB1, and ABCG2 along with increased proliferation (Figure 3C). When adherent cells and spheres of A2780 cells were treated with CoCl_2_, a chemical stabilizing HIF-1α, the expression levels of ABCB1 and ABCG2 were time-dependently increased up to 48 h (Supplementary Figure S5). Immunocytochemistry analysis of spheres showed increased expression of SOX2 inside spheres (Figure 3D). Consistent with the hypoxia-induced expression of ALDH1, flow cytometry analysis of sphere cells consistently showed increased activity of ALDH in response to hypoxia treatment (Figure 3E). These results suggest that hypoxic treatment of ovarian cancer cells promotes CSC-like characteristics.

### NOTCH1 signaling is activated in ovarian CSCs

**Figure 4:**
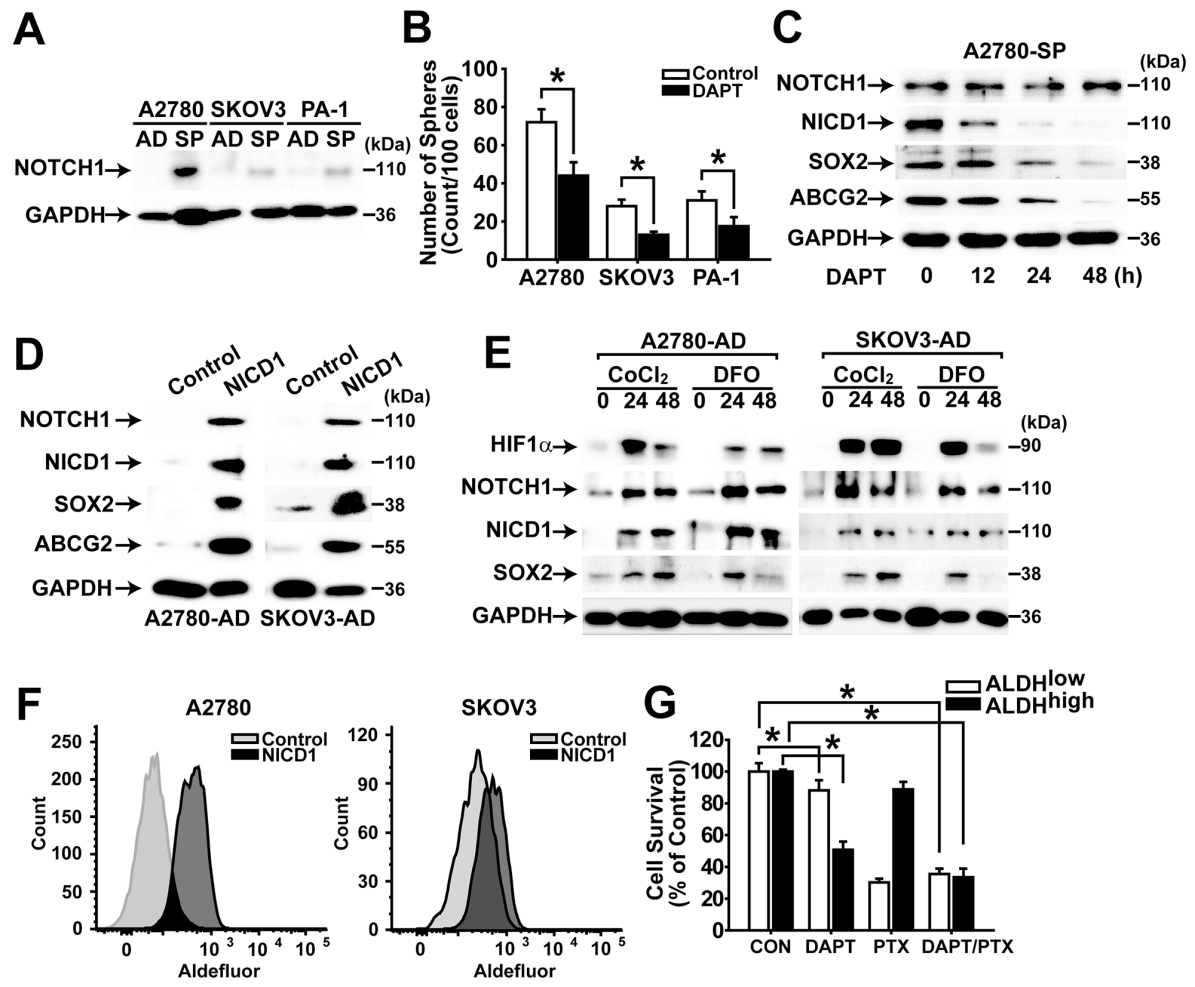
NOTCH1 signaling enhances CSC properties in ovarian cancer cells **A.** Western blotting analysis results of adherent cells (AD) or spheres (SP) of A2780, SKOV3, and PA-1 cells with indicated probes are shown. **B.** Numbers of spheres from A2780, SKOV3, and PA-1 cells with or without NOTCH inhibitor (DAPT, 10 μM) are shown. Data indicate mean ± SD (n=4). *, P<0.05. **C.** Western blotting analysis results of A2780 sphere cells after incubation with DAPT (20 μM) for 0, 12, 24, or 48 h are shown with indicated probing antibodies. **D.** Western blotting analysis results of adherent A2780 and SKOV3 cells with or without NICD1 overexpression are shown with indicated probing antibodies. **E.** Western blotting analysis results of adherent A2780 and SKOV3 cells after incubation with CoCl_2_ (100 μM) or DFO (200 μM) for 0, 24, or 48h are shown with indicated probing antibodies. **F.** Flow cytometry analysis results of adherent A2780 and SKOV3 cells with or without NICD1 overexpression after incubation with Aldefluor for measuring ALDH activity are shown. **G.** Viability of ALDH^high^ and ALDH^low^ populations from adherent A2780 cells after incubation of DAPT (10 μM) and/or paclitaxel (1 μM) for 48 h was determined by MTT assay. The percentage of viable cells is shown after normalization to no treatment control. Data indicate mean ± SD (n=4). *, P<0.05.

NOTCH signaling is known to be involved in self-renewal of stem cells [13, 14]. To clarify the role of NOTCH signaling in ovarian cancer cells, the expression levels of NOTCH1 and NICD1 were determined in adherent cells and spheres of A2780, SKOV3, and PA-1 cells. Spheres isolated from A2780, SKOV3, and PA-1 cells showed the higher expression of NOTCH1 compared with their adherent cells, and inhibition of NOTCH signaling by DAPT treatment resulted in significantly decreased sphere forming ability (Figure 4A and 4B). DAPT treatment of spheres from A2780 and SKOV3 cells exhibited time-dependent decrease of NICD1 generation, and the expression levels of SOX2 and ABCG2 decreased accordingly (Figure 4C and Supplementary Figure 6A). Overexpression of NICD1 in adherent A2780 and SKOV3 cells resulted in increased expression of NOTCH1, SOX2, and ABCG2 (Figure 4D). When hypoxia-mimicking conditions were induced by CoCl_2_ or deferoxamine (DFO) treatment in A2780 or SKOV3 cells, HIF-1α expression was induced and expression levels of NOTCH1 and SOX2 increased along with increased generation of NICD1 (Figure 4E). Overexpression of NICD1 in A2780 or SKOV3 adherent cells resulted in significantly increased ALDH activity (Figure 4F). When ALDH^high^ cells and ALDH^low^ cells were sorted and treated with DAPT or paclitaxel, DAPT treatment resulted in decreased viability of ALDH^high^ cells, whereas paclitaxel treatment decreased viability of ALDH^low^ cells (Figure 4G). Co-treatment of DAPT and paclitaxel resulted in decreased viability of both ALDH^high^ cells and ALDH^low^ (Figure 4G). These results suggest that NOTCH1 signaling is critical for maintaining and inducing CSC characteristics in ovarian cancer cells. In addition, NOTCH1 signaling may mediate hypoxia signaling to induce SOX2 expression, and co-treatment with DAPT and paclitaxel resulted in decreased viability of both CSC- and non-CSC populations in ovarian cancer cells.

### Hypoxia increases SOX2 promoter activity through NOTCH1 activation

**Figure 5:**
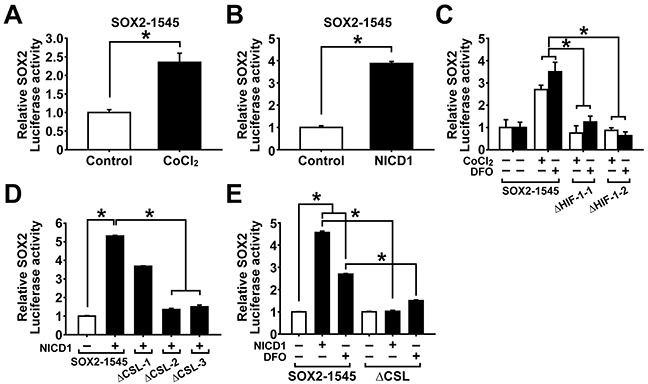
Hypoxia and NOTCH1 signaling increase SOX2 promoter activity **A.** SOX promoter activity in adherent A2780 cells after CoCl_2_ treatment (100 μM, 48 h) is shown. Data indicate mean ± SD (n=4). *, P<0.05. **B.** SOX promoter activity in adherent A2780 cells with or without NICD1 overexpression is shown. Data indicate mean ± SD (n=4). *, P<0.05. **C.** SOX2 promoter activities of wild type construct or HIF-1-deletion constructs (ΔHIF-1-1, ΔHIF-1-2) in adherent A2780 cells after CoCl_2_ (100 μM, 48 h) or DFO (200 μM, 48 h) treatment are shown. Data indicate mean ± SD (n=4). *, P<0.05. **D.** SOX2 promoter activities of wild type construct or CSL binding sites (1-3)-deletion construct (ΔCSL) in adherent A2780 cells with or without NICD1 overexpression or DFO treatment (200 μM, 48 h) are shown. Data indicate mean ± SD (n=4). *, P<0.05. **E.** SOX2 promoter activities of wild type construct or individual CSL-binding site-deletion constructs (ΔCSL-1, ΔCSL-2, ΔCSL-3) in adherent A2780 cells with or without NICD1 overexpression are shown. Data indicate mean ± SD (n=4). *, P<0.05.

We observed that expression of SOX2 was significantly increased by hypoxic stress or NICD1 overexpression. To explore whether hypoxic culture condition can activate SOX2 transcription, A2780 ovarian cancer cells were transfected with SOX2 promoter reporter construct and treated with CoCl_2_. As shown in Figure 5A, CoCl_2_ treatment resulted in significantly increased SOX2 promoter activity (SOX2-1545). To evaluate whether NOTCH1 signaling can activate SOX2 transcription, A2780 ovarian cancer cells were co-transfected with SOX2 promoter-driven reporter construct and NICD1 overexpression construct. As shown in Figure 5B, NICD1 overexpression resulted in significantly increased SOX2 promoter activity. When binding sites of HIF-1α and CSL, a DNA binding partner of NOTCH1 signaling, were searched in the SOX2 promoter reporter construct, two HIF-1 binding sites and three CSL bindings sites were identified (Supplementary Figure S7). Individual deletion of HIF-1 binding sites in the SOX2 promoter reporter construct resulted in a significant decrease of CoCl_2_- or DFO-induced activation of SOX2 promoter (Figure 5C). To evaluate whether hypoxia-induced activation of SOX2 promoter activity is mediated by NOTCH1 activation, A2780 ovarian cancer cells were transfected with wild type SOX2 promoter reporter construct or CSL binding sites (1-3)-deletion construct with or without NICD1 overexpression or DFO treatment. As shown in Figure 5D, deletion of individual CSL binding site showed that CSL-2 and CSL-3 are required for NICD1-induced activation of SOX2 promoter activity. NICD1 overexpression or DFO treatment alone increased wild type SOX2 promoter activity. Deletion of CSL binding sites in SOX2 promoter decreased NICD1-induced activation of SOX2 promoter as well as DFO-induced activation of SOX2 promoter (Figure 5E). These results suggest that SOX2 transcription is stimulated by hypoxia and NOTCH1 signaling, in which hypoxic signaling is mediated by NOTCH1 signaling in ovarian cancer cells.

### SOX2 expression is required for hypoxia- or NOTCH1-induced acquisition of CSC-like characteristics in ovarian cancer cells

**Figure 6:**
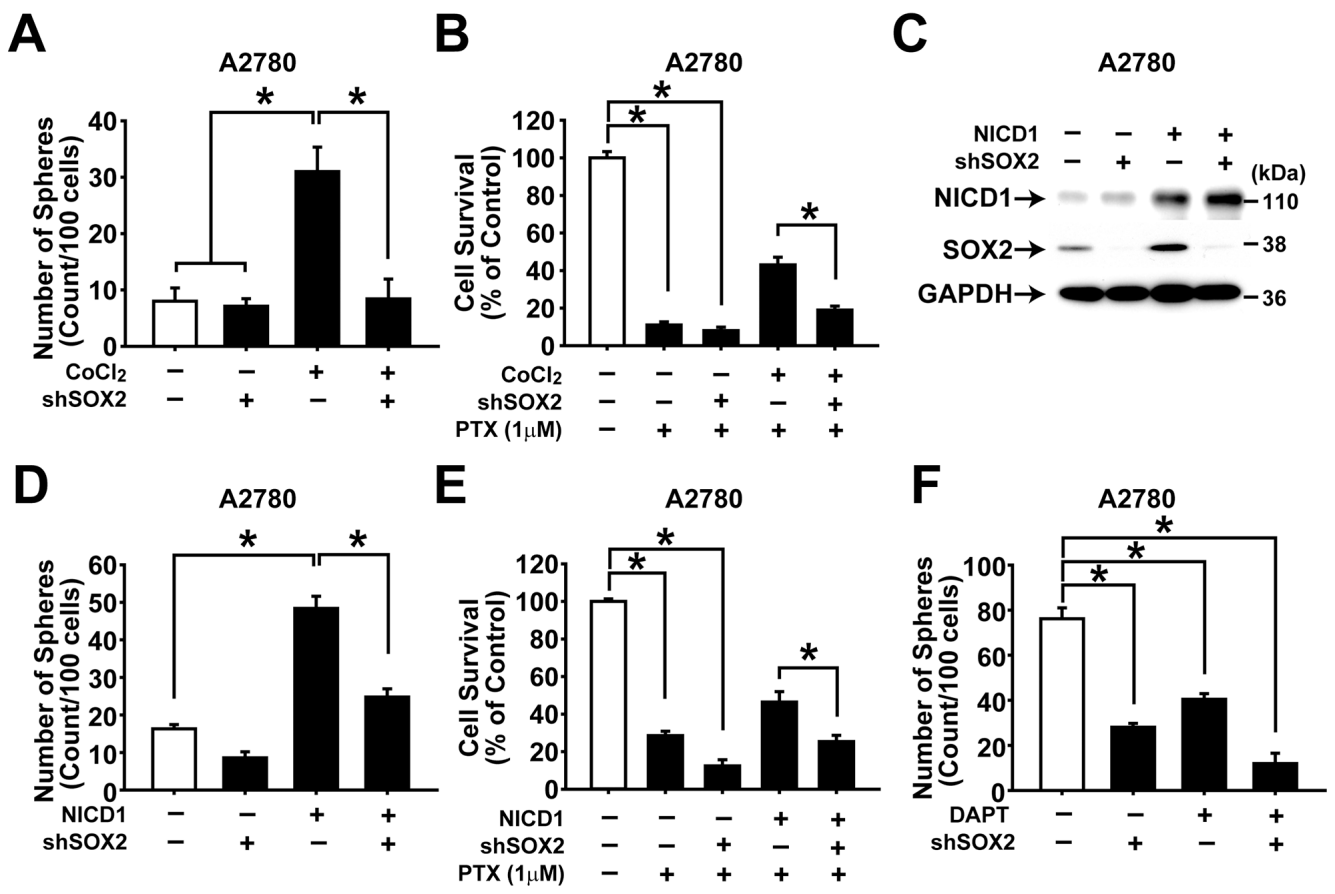
NOTCH1 and SOX2 are important for maintaining CSC properties in ovarian cancer cells **A.** Numbers of spheres generated from adherent A2780 cells with or without CoCl_2_ (100 μM) treatment in combination with SOX2 knockdown are shown. The numbers of spheres were counted on day 7 after treatment. Data indicate mean ± SD (n=4). *, P<0.05. **B.** Viability of adherent A2780 cells with or without paclitaxel (1 μM) treatment was determined by MTT assay. Paclitaxel was treated for two days in combination with CoCl_2_ (100 μM) and/or SOX2 knockdown. The percentage of viable cells is shown after normalization to no treatment control. Data indicate mean ± SD (n=4). *, P<0.05. **C.** Western blotting analysis results of adherent A2780 cells with NICD1 overexpression in combination with SOX2 knockdown are shown with indicated probing antibodies. **D.** Numbers of spheres generated from adherent A2780 cells with NICD1 overexpression in combination with SOX2 knockdown are shown. The numbers of spheres were counted after 7 days. Data indicate mean ± SD (n=4). *, P<0.05. **E.** Viability of adherent A2780 cells with or without paclitaxel (1 μM) treatment was determined by MTT assay. Paclitaxel was treated for two days in combination with NICD1 overexpression and SOX2 knockdown. The percentage of viable cells is shown after normalization to no treatment control. Data indicate mean ± SD (n=4). *, P<0.05. **F.** Numbers of spheres generated from adherent A2780 cells with DAPT treatment (10 μM, 48 h) in combination with SOX2 knockdown are shown. Data indicate mean ± SD (n=4). *, P<0.05.

To explore whether SOX2 can mediate hypoxia- or NOTCH1-induced augmentation of CSC characteristics in ovarian cancer cells, SOX2 expression was silenced together with incubation with hypoxic condition or NICD1 overexpression in A2780 and SKOV3 ovarian cancer cells. Knockdown of SOX2 expression with shRNA resulted in significantly decreased CoCl_2_-induced augmentation of sphere forming activity and resistance to paclitaxel (Figure 6A and 6B and Supplementary Figure S6B and S6C). Moreover, knockdown of SOX2 expression along with NICD overexpression resulted in significantly decreased NICD1-induced augmentation of sphere forming activity or resistance to paclitaxel in A2780 (Figure 6C–6E and Supplementary Figure S6D and S6E). DAPT treatment, SOX2 knockdown, and combination of DAPT treatment and SOX2 knockdown decreased sphere forming activity of A2780 ovarian cancer cells (Figure 6F). To further confirm the role of hypoxia-NOTCH1-SOX2 signaling axis in ovarian CSCs, A2780 cells were treated with different combinations of CoCl_2_, DAPT, and SOX2 overexpression. As shown in Supplementary Figure S8A, DAPT treatment markedly attenuated CoCl_2_-induced increase in the sphere forming activity. SOX2 overexpression, however, abolished the inhibition of DAPT on the sphere forming activity. When drug resistance was measured, DAPT treatment inhibited CoCl_2_ induction of the drug resistance in A2780 cells. SOX2 overexpression, however, abolished the DAPT-induced inhibition of the drug resistance, whereas SOX2 knockdown did not (Supplementary Figure S8B). These results suggest that the hypoxia-NOTCH1-SOX2 signaling axis is critical for acquisition of CSC characteristics in ovarian cancer cells.

### SOX2 is important for maintaining drug resistance of primary ovarian CSCs

**Figure 7:**
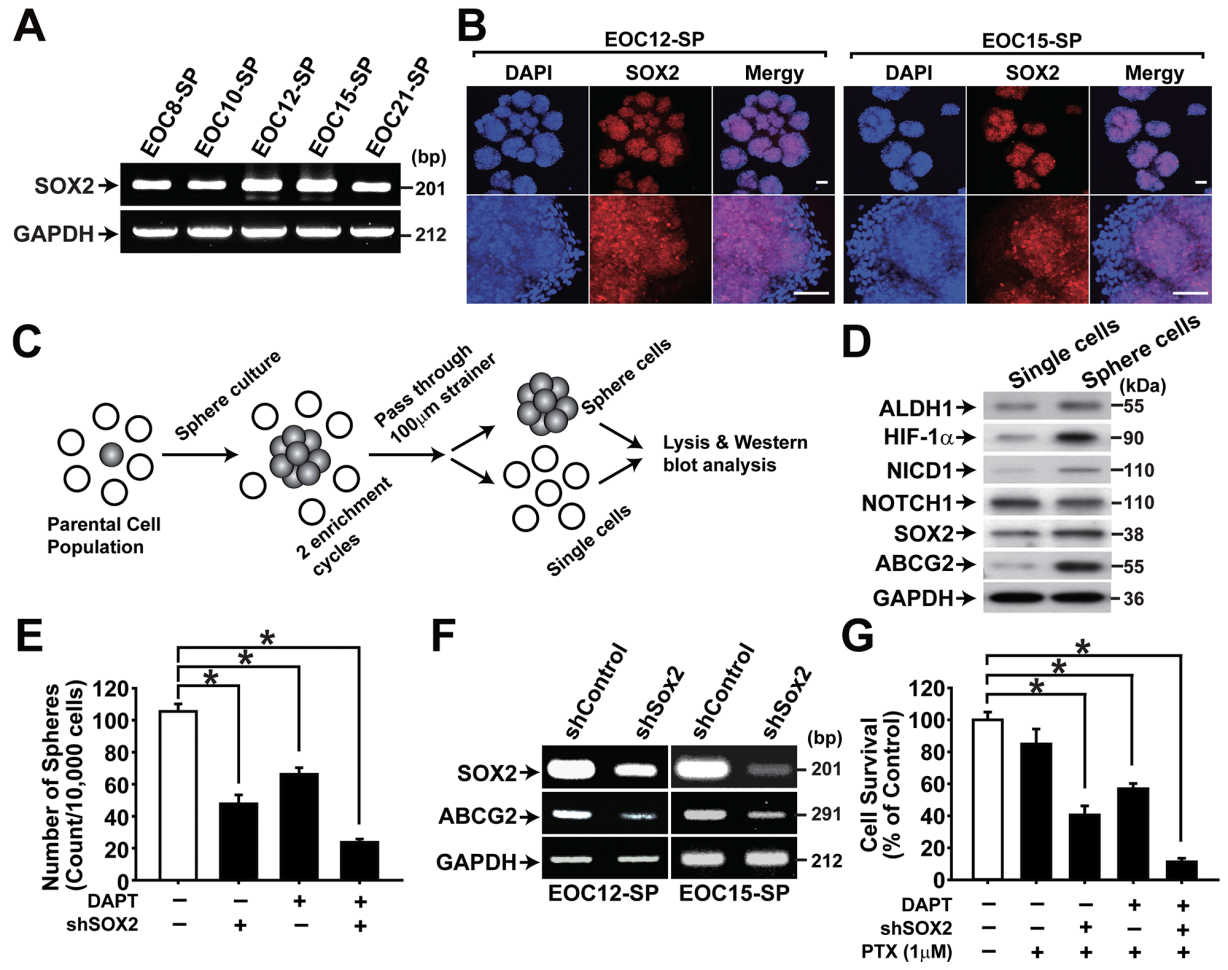
NOTCH1 and SOX2 are important for maintaining CSC properties in primary ovarian cancer cells **A.** RT-PCR results of spheres generated from primary epithelial ovarian cancer cells are shown with indicated probes. **B.** Confocal microscopy images of spheres generated from EOC-12 (left panels) and EOC-15 (right panels) after immunolabeling with anti-SOX2 antibody. Nuclei were stained with DAPI. Scale bar = 100 μm. **C.** Schematic outline of the sphere enrichment protocol for primary EOC culture (100 μm < sphere size). **D.** Western blotting analysis results of single cells and spheres generated from EOC-12 are shown with indicated probing antibodies. **E.** Sphere numbers generated from ECS-12 with or with DAPT treatment (10 μM, 48 h) in combination with SOX2 knockdown are shown. Data indicate mean ± SD (n=4). *, P<0.05. **F.** RT-PCR results of spheres generated from ECO-12 (left panels) and EOC15 (right panels) with or without SOX2 knockdown are shown with indicated probes. **G.** Viability of sphere cells from EOC-12 with or without paclitaxel (1 μM) treatment was determined by MTT assay. Paclitaxel was treated for two days in combination with DAPT treatment (10 μM) and SOX2 knockdown. The percentage of viable cells is shown after normalization to no treatment control. Data indicate mean ± SD (n=4). *, P<0.05.

CSC-enriched spheres were isolated from five different primary ovarian cancer tissues. Spheres from primary ovarian cancer tissues showed high expression of SOX2 (Figure 7A and 7B). When single cells and spheres were isolated from primary ovarian sphere cultures, spheres showed the higher expression of stemness-related markers ALDH1, SOX2, and ABCG2 along with the higher expression of HIF-1α and NICD1 (Figure 7C and 7D). Knockdown of SOX2 expression with shRNA in primary ovarian cancer spheres resulted in significantly decreased sphere forming activity and ABCG2 expression (Figure 7E and 7F). DAPT treatment, SOX2 knockdown, and combination of DAPT treatment and SOX2 knockdown decreased resistance to paclitaxel (Figure 7G). These results suggest that SOX2 increases CSC characteristics and drug resistance in primary ovarian CSCs.

## DISCUSSION

Involvement of hypoxia in the activation of stemness-related gene expression in cancer cells has been reported [30]. HIFs have been shown to play important roles in specifying and maintaining CSCs in glioblastoma, leukemia, and breast cancer [31–33]. In the present study, we demonstrated that culturing CSC-enriched spheres in hypoxic condition further enhanced CSC-characteristics including sphere size, expression of SOX2 and ALDH1, and cell proliferation. In support of the results, hypoxic pretreatment of ovarian cancer cells, ES-2 and OVCAR-3, increased expression of OCT4 and SOX2 and stem-like properties including sphere formation, high proliferation, and high infiltration [12]. These results indicate that hypoxia induces CSC-characteristics in ovarian cancer cells.

Hypoxia is known to fully activate expression of NOTCH target genes through induction of HIF-1α, which joins transcription activation complex including NOTCH intracellular domain [14]. NOTCH shows oncogenic effects in certain tumors, and the contribution of NOTCH to CSC formation has been suggested [34]. Gene expression analysis of CSC-enriched ovarian cancer populations, including sphere cells, side population, and high-grade serous ovarian cancer, showed increased expression of NOTCH1 [35–37]. In glioblastoma stem cells, hypoxia stimulates HIF-correlated NOTCH signaling [38]. In our study, hypoxic treatment of ovarian cancer cells resulted in increased expression of NOTCH1 and generation of NICD1. In addition, CSC-enriched spheres from different ovarian cancer cells showed increased expression of NOTCH1, and inhibition of NOTCH signaling with DAPT resulted in loss of CSC characteristics, whereas NICD1 overexpression led to acquisition of CSC characteristics. In addition, hypoxia-induced stimulation of SOX2 promoter activity was abolished by deletion of CSL binding sites in the SOX2 promoter region. In a previous study, overexpression of NOTCH3 in ovarian cancer cells promoted expansion of CSCs, and treatment with γ–secretase inhibitor resulted in depletion of CSCs with increased sensitivity to platinum drug and decreased expression of stemness-related gene expression [39, 40]. Taken together, our results suggest the importance of NOTCH signaling for maintaining hypoxia-induced CSC characteristics in ovarian cancer cells.

SOX2 expression has been detected in various cancers [24]. In gastric cancer and non-small cell lung cancer, siRNA-mediated SOX2 knockdown resulted in decreased sphere-forming ability and self-renewal of the CSC population [41, 42]. In human serous ovarian cancer cells, SOX2 overexpression increased CSC marker expression, sphere formation, and *in vivo* tumorigenic activity, with little effect on cell proliferation [25]. In our study, SOX2 was a critical downstream effector of hypoxia- and NICD1-induced enhancement of CSC characteristics. SOX2 promoter activity was directly stimulated by HIF-1α and NICD1, and hypoxia- or NICD1-induced augmentation of CSC characteristics was abolished by knockdown of SOX2 expression. The interaction between Notch1 and Sox2 was suggested through direct activation of Sox2 transcription by NICD-CSL [43]. Furthermore, CSL-dependent activation of Sox2 after ectopic expression of NICD was also demonstrated [44]. In the future study, chromatin immunoprecipitation analysis with HIF-1α or NICD1 antibodies would complement the current results. Taken together, the results suggest that SOX2 is a critical mediator of NOTCH signaling for maintaining stemness-related characteristics in ovarian CSCs.

In conclusion, we show that CSC characteristics in not only ovarian cancer cell lines but also primary CSCs are augmented in hypoxic condition, and NOTCH1 and SOX2 are important mediators of hypoxia in ovarian CSCs. In addition, NOTCH signaling was necessary for hypoxia-induced augmentation of CSC characteristics and SOX2 was necessary for hypoxia- or NICD-induced augmentation of CSC characteristics in ovarian cancer cells. There results suggest that hypoxia-NOTCH1-SOX2 signaling axis is an important for maintaining CSCs in ovarian cancer, which may provide a novel opportunity for development of drugs to eliminate CSCs in ovarian cancer patients.

## MATERIALS AND METHODS

### Materials

RPMI1640 medium was purchased from Welgene (Gyeongsan, South Korea). HBSS (Hank's Balanced Salt Solution), trypsin, fetal bovine serum (FBS), B-27 Supplement, penicillin, streptomycin, and Accutase cell detachment solution, and Lipofectamine/ Lipofectamine Plus reagent were purchased from Life Technologies (Grand Island, NY). Basic fibroblast growth factor (bFGF) and epidermal growth factor (EGF) were purchased from R&D Systems (Minneapolis, MN). Cell culture plates for adherent cells were purchased from Thermo Fisher Scientific Inc. (Waltham, MA). For culture of sphere cells, culture plates with ultra-low attachment surface were purchased from Corning Life Sciences (Tewksbury, MA). Human ovarian cancer cell lines SKOV3 and PA-1 were purchased from the American Type Culture Collection (Manassas, VA). Antibodies against glyceraldehyde-3-phosphate Dehydrogenase (GAPDH) were purchased from EMD Millipore (Billerica, MA). Antibodies against HIF-1α (#610958), ALDH1 (#611194) were purchased from BD Biosciences (San Jose, CA). Antibodies against SOX2 (sc-17320) and secondary antibodies conjugated to HRP were purchased from Santa Cruz Biotechnology. Antibodies against ABCG2 (ab3380) were purchased from Abcam (Boston, MA). Antibodies against ABCB1 (#12683), NOTCH1 (#4380), and Cleaved NOTCH1 (#4147) were purchased from Cell Signaling Technology (Danvers, MA). Human ovarian cancer cell line A2780, cobalt chloride (CoCl_2_), deferoxamine mesylate/Desferal (DFO), dimethyl sulfoxide, laminin, amphotericin, bisBenzimide H33258 (#14530), Ponceau S solution, and all other chemicals, unless otherwise stated, were obtained from Sigma–Aldrich (Saint Louis, MO).

### Cell culture

Human ovarian cancer cell lines, A2780, SKOV3, and PA-1, were grown in the culture medium and culture conditions recommended by the ATCC and harvested by treatment with 0.25% trypsin. To generate spheres of cells, over-confluent cultures of A2780, SKOV3, or PA-1 cells were switched from 10% FBS to 1% FBS in RPMI-1640 or α-MEM media. After two days, floating spheres were collected by centrifugation of the supernatant at 800 rpm (Hanil FLETA 5, rotor HSR-4S). Harvested spheres (A2780-SP, SKOV3-SP, and PA-1-SP) were grown in sphere culture medium (Neurobasal media (1×) (Life Technologies) supplemented with 20 ng/mL bFGF, 10 ng/mL EGF, 2.5 μg/mL amphotericin, 100 IU/mL penicillin, 100 μg/mL streptomycin, and B-27 Supplement (50×) (Life Technologies, without serum) in Ultra-Low Attachment six-well plates. Fresh medium was added every two or three days. Spheres were transferred to the next generation by dissociation into single cells with Accutase followed by filtering through a 40-μm cell strainer and plating at 10^4^ cells/mL. Third-generation spheres were used for all subsequent experiments.

### Sphere formation assay

Cells were plated in Corning Ultra-Low Attachment 24-well plates at a density of 100-10,000 viable cells/well and grown in sphere culture medium or corresponding experimental medium. Cells were grown for 7 days, and the numbers of spheres were counted under a microscope.

### Cell migration assay

Ovarian cancer cells were harvested with 0.05% trypsin containing 0.02% EDTA, and suspended in a RPMI medium with 10% FBS at a concentration of 1×10^5^ cells/mL. Membrane filters (8-μm pore size) in disposable 96-well chemotaxis chambers (Neuro Probe, Gaithersburg, MD) were pre-coated for 6 h with 20 μg/mL rat-tail collagen at room temperature. Aliquots (50 μL per well) of the cell suspension were loaded into the upper chambers, and RPMI medium with 10% FBS or experimental medium was placed in the lower chamber. After incubation for 12 h at 37°C, the filters were disassembled, and the upper surface of each filter was scraped free of cells by wiping with a cotton swab. The numbers of cells that had migrated to the lower surfaces of each filter were determined by counting the cells in three different places under the microscope (×100 magnification) after staining with Hoechst 33342 (10 μM).

### Hypoxic treatment

For hypoxia treatments, A2780 cells were incubated under hypoxic conditions with 1% O_2_ using a Modular Incubator Chamber MIC-101 (Billups-Rothenberg Inc., CA) or in the presence of 100 μM DFO and CoCl_2_ for 24 h. Sphere cells were plated into laminin-coated 6-well tissue plates (1×10^5^/well), and CoCl_2_ and DFO stock solutions were added to cell cultures to a final concentration of 100 μM. After incubation at 37°C for the indicated time periods, cell culture medium was removed, and cells were harvested after washing with HBSS solution

### Flow cytometric analysis

Aldehyde dehydrogenase (ALDH) activity was measured using the Aldefluor assay Kit (STEMCELL Technologies, Vancouver, BC, Canada) as described by the manufacturer. Briefly, dissociated single cells from adherent or sphere cells were re-suspended in Aldefluor assay buffer containing an ALDH substrate, bodipy-aminoacetaldehyde (BAAA), at 7.5 μM, and incubated for 1 hr at 37°C. An identical reaction was also performed in the presence of 15 mM diethylaminobenzaldehyde (DEAB), an ALDH-specific inhibitor. Analysis of fluorescence intensity of the stained cells was performed using a FACSAria-III cell sorter (BD Biosciences, Franklin Lake, NJ). ALDH activity of the sample was determined based on the fluorescence intensity beyond the threshold defined by the reaction with DEAB. Aldefluor staining was detected using the FITC channel. To prevent cross-contamination between ALDH^high^ and ALDH^low^ cells, sorting gates of these two populations were set up at least one log apart. The purity of the sorted populations was reanalyzed using ALDH^high^ and ALDH^low^ cells and was found to be greater than 95%.

### Analysis of drug-induced cell death

A2780 spheres (A2780-SP) cells were dissociated into single cells by treatment with Accutase, filtered through a 40-μm cell strainer, and seeded at 10,000 single cells per well into 24-well Ultra-Low Attachment plates (Corning) in 500 μL sphere culture medium. In parallel, A2780 cells were seeded at 10,000 cells per well in RPMI-1640 media with 10% FBS into tissue culture 24-well plates (Corning). After 24 h, A2780-SP and A2780 cells were switched to Neurobasal media without supplements or serum or RPMI-1640 media, respectively, followed by addition of paclitaxel (Sigma-Aldrich). After treatment with paclitaxel for 48 h, cells were harvested and subjected to the MTT (3-(4,5-dimethylthiazol-2-yl)-2,5-diphenyltetrazolium bromide) assay. For the MTT assay, cells were washed twice with PBS and incubated with 100 μL of MTT (0.5 mg/mL) for 2 h at 37°C. Formazan granules generated by the cells were dissolved in 100 μL of dimethyl sulfoxide, and the absorbance of the solution at 562 nm was determined using a Sunrise^TM^ Absorbance Reader (Tecan Trading AG, Switzerland) after dilution to a linear range.

### Gene silencing using shRNA lentivirus and production of retroviruses

pLKO.1-puro lentiviral vectors expressing SOX2 shRNA and control shRNA (SHC002) were purchased from Sigma-Aldrich. For generation of lentiviral particles, HEK293FT cells were co-transfected with the shRNA lentiviral plasmid (pLKO.1-puro) and ViraPower Lentiviral packaging mix (pLP1, pLP2, pLP-VSV-G; Life Technologies) using Lipofectamine 2000 and the culture supernatants containing lentivirus were harvested at 48 h after transfection. For lentiviral transduction, cells were treated with the shRNA-expressing lentivirus in the presence of 5 μg/mL polybrene (Sigma-Aldrich, MO). To ensure shRNA-mediated silencing of SOX2 expression, the mRNA levels of SOX2 and GAPDH were determined by RT-PCR analysis.

Retroviral plasmids carrying the pMX-SOX2 were kindly provided by Jeong Beom Kim (UNIST, Republic of Korea) and individually co-transfected with packaging plasmids (gag-pol and VSV-G) into Plat-GP cells using Lipofectamine-Plus reagent. Plat-GP cells (RV-103, Cell Biolabs, Inc., San Diego, CA) were maintained in DMEM with high glucose, 10% FBS, 10 μg/mL blasticidin, and 1× penicillin-streptomycin. Virus-containing supernatants were collected two days after transfection, passed through a 0.45 μm filter, and stored at −80°C.

### Preparation of reporter constructs and luciferase assay

The human SOX2 promoter containing the HIF1- and CSL-binding elements luciferase report plasmids were cloned by PCR of human genomic DNA (forward: 5′-GGAAGGAAACTTAGACGAGGC-3′; reverse: 5′- CTTCTCTCCCTTTCTTTCTC-3′ for SOX2) and excising the promoter fragment from the above mentioned products and subcloned into the Kpn1 (NEB #R3142) and HindIII (NEB #R3104) site of the pGL3 basic vector (Promega, Madison, WI). ABCG2 promoter construct was a kind donation from Dr Ross [45]. NANOG-Core promoter was purchased from Addgene (pNANOG-Luc; Plasmid #25900). Deletion sequences in reporter constructs are as follows: human ABCG2 promoter (TTGATTTGTTTTTACTT, TGGAAATGTTTTCATTT); human OCT4 promoter (ATCTAAAAACAAGAGGG); human SOX2 promoter (TAGCGACAACAAGAGAA). Deletion mutant constructs were generated using the QuikChange II XL Site-Directed Mutagenesis Kit (Agilent Technologies) according to the manufacture's instruction. The intended mutations were confirmed by sequencing analysis of constructs.

### Western blotting analysis

For Western blotting analysis, cells were harvested and lysed in lysis buffer (20 mM Tris-HCl, 1 mM EGTA, 1 mM EDTA, 10 mM NaCl, 0.1 mM phenylmethylsulfonyl fluoride, 1 mM Na_3_VO_4_, 30 mM sodium pyrophosphate, 25 mM β-glycerol phosphate, and 1% Triton X-100; pH 7.4). Lysates were resolved by SDS-PAGE, transferred to a nitrocellulose membrane, and then stained with 0.1% Ponceau S solution. After blocking in 5% nonfat milk, the membranes were immunoblotted with various antibodies, and the bound antibodies were visualized with horseradish peroxidase-conjugated secondary antibodies using the enhanced chemiluminescence Western blotting kit (ECL, Amersham Biosciences).

### RNA extraction and RT-PCR

Total RNA was extracted from 80–90% confluent cultures using TRIzol reagent (Sigma-Aldrich) and reverse transcribed into cDNA using the Reverse Transcription cDNA Kit (#RT50KN; NanoHelix Co., Ltd). cDNA in 1 μL of the reaction mixture was amplified using the Ready-2×-Go pre-mix PCR kit (#PMD008L; NanoHelix) and 10 pmol each of sense and antisense primers. The thermal cycle profile was as follows: denaturation at 95°C for 30 s, annealing at 54°C for 30 s depending on the primers used, and extension at 72°C for 30 s. Each PCR reaction was carried out for 25-30 cycles and PCR products were analyzed by 1% agarose gel electrophoresis. The following primer pairs were used: SOX2: 5′-CAACATGATGGAGACGGAGC-3′, 5′-GTG CATCTTGGGGTTCTCCT-3′; ALDH1: 5′-CTCGAAA TTAAGTACACCAA-3′, 5′-TCAGTAGA CCCTGTGAA TGC-3′; ABCB1: 5′-CCATCAGTCCTGTTCTTG-3′, 5′-CTGCTCCTCTTGCATTT-3′; ABCG2: 5′-TTCAGCC GTGGAACTCTTTG-3′, 5′-CCACACTCTGACCTGCTG CT-3′; GAPDH: 5′-TCACCATCTTCCAGGAGCG-3′, 5′-CTGCTTCACCACCTTCTTGA-3′.

### Immunofluorescence staining

For immunofluorescence staining, cells were fixed in 4% paraformaldehyde in PBS for 10 min, washed twice with PBS, and blocked with 1% FBS in PBS for 30 min; all procedures were performed at room temperature. The fixed specimens were incubated with primary antibodies for 1 h, followed by incubation with secondary antibodies for 1 h. Primary antibodies (1:100) were detected by Alexa Fluor 488 and Alexa Fluor 568 conjugated secondary antibodies (1:1000) (Life Technologies). The specimens were finally washed and mounted in Vectashield medium (Vector Laboratories, Burlingame, CA) with 4′,6-diamidino-2-phenylindole for visualization of nuclei. The stained sections were visualized using laser scanning confocal microscopy (Olympus FluoView FV1000).

### Statistical analysis

Data are expressed as mean ± S.E. for *in vitro* studies. Statistical significance (*, P<0.05) was determined using two tailed unpaired t-tests. Unless stated otherwise, all experiments were performed in triplicate.

## SUPPLEMENTARY FIGURES



## Abbreviations

ALDH: aldehyde dehydrogenase
CSCs: cancer stem cells
EOC: epithelial ovarian cancer
HIF: hypoxia-inducible factor
NICD1: intracellular domain of NOTCH1
