# Supplementary material for: Hypoxia-NOTCH1-SOX2 signaling is important for maintaining cancer stem cells in ovarian cancer
Source: Oncotarget. 2016 Jul 30;7(34):55624–38. doi: 10.18632/oncotarget.10954 (PMC5342441; doi:10.18632/oncotarget.10954)
Supplement: Supplementary file 1 [file oncotarget-07-55624-s001.pdf]

Hypoxia-NOTCH1-SOX2 signaling is important for maintaining cancer stem cells in ovarian cancer

SUPPLEMENTARY FIGURES

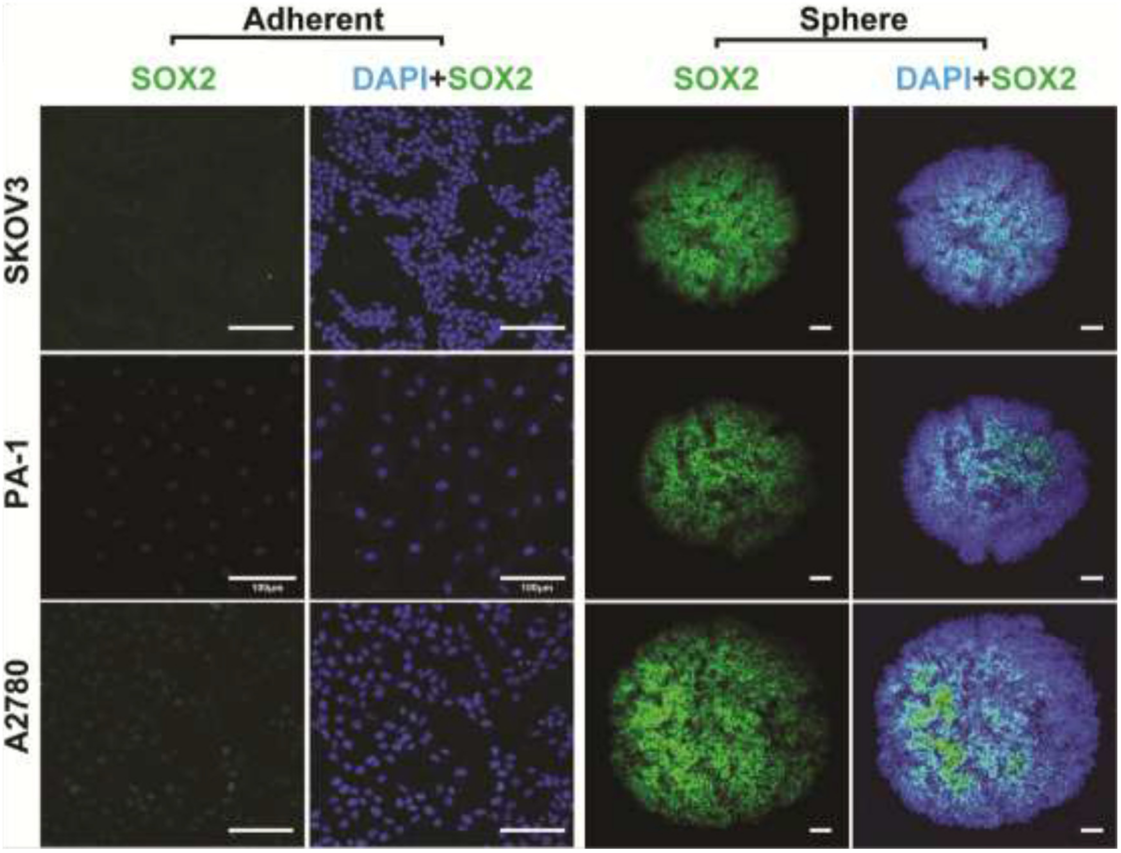

Supplementary Figure S1: SOX2 expression is increased in spheres of ovarian cancer cells. Confocal images of adherent cells (left panels) and spheres (right panels) from SKOV3, PA-1, and A2780 ovarian cancer cells after immunolabeling with anti-SOX2 antibody are shown. Nuclei were stained with DAPI. Scale bar=100 µm.

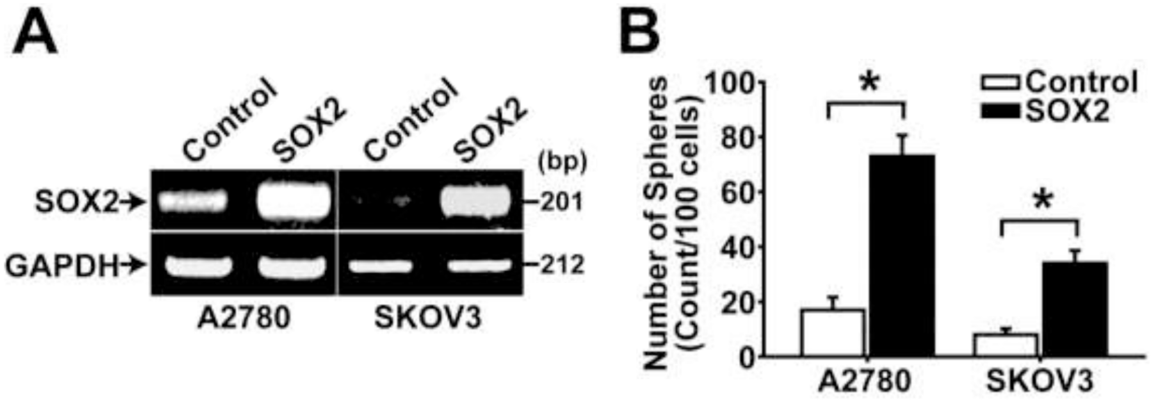

Supplementary Figure S2: SOX2 overexpression stimulates sphere formation of ovarian cancer cells. A. RT-PCR results of adherent A2780 and SKOV3 cells with or without SOX2 overexpression are shown with indicated probes. **B.** Numbers of spheres generated from adherent A2780 and SKOV3 cells with or without SOX2 overexpression are shown. Data indicate mean ± SD (n=3). *, P<0.05.

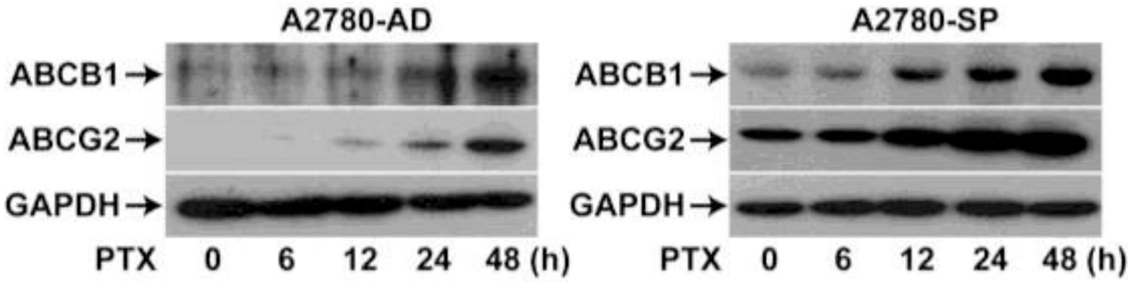

Supplementary Figure S3: Paclitaxel treatment increases the expression of ABC trasnporters. Western blot analysis results of adherent cells (AD) and spheres (SP) of A2780 cells after incubation with paclitaxel (PTX) (1 μM) for indicated hours are shown.

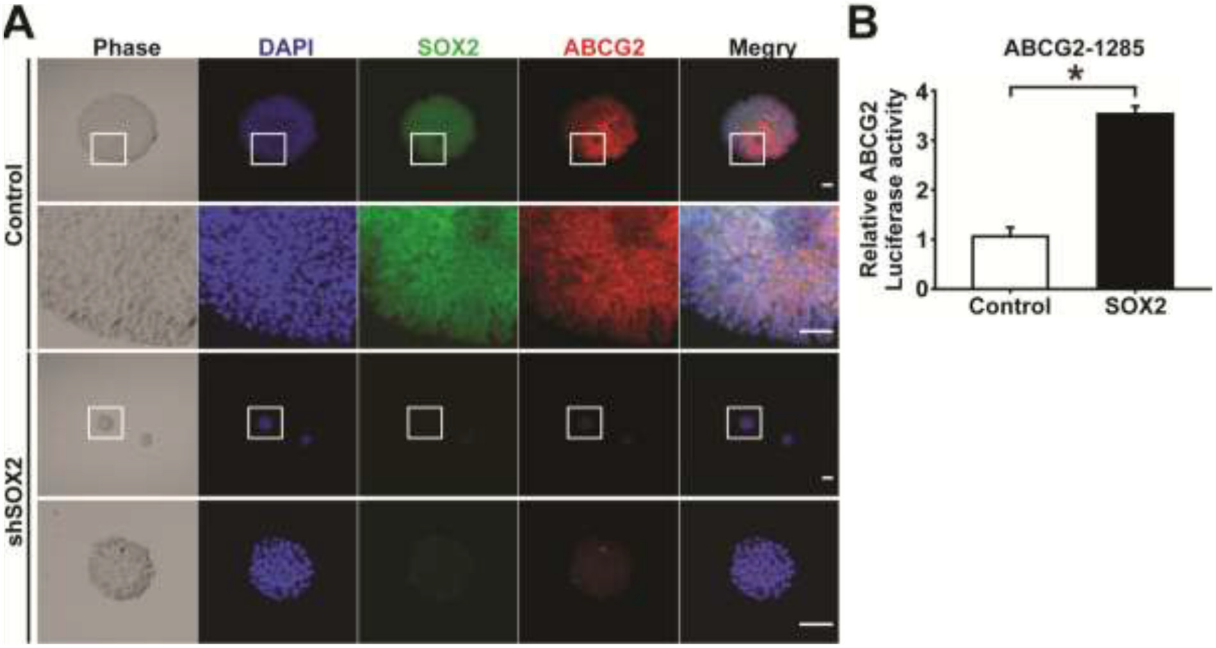

Supplementary Figure S4: SOX2 knockdown decreases ABCG2 expression and SOX2 overexpression increases ABCG2 promoter activity. A. Confocal microscope images of A2780 spheres with or without SOX2 knockdown are shown after immunolabeling with anti-SOX2 antibody or anti-ABCG2 antibody. Nuclei were stained with DAPI. Scale bar = 100 µm. **B.** Promoter activities of ABCG2 with or without SOX2 overexpression in adherent A280 cells are shown. Data indicate mean ± SD (n=3). *, P<0.05.

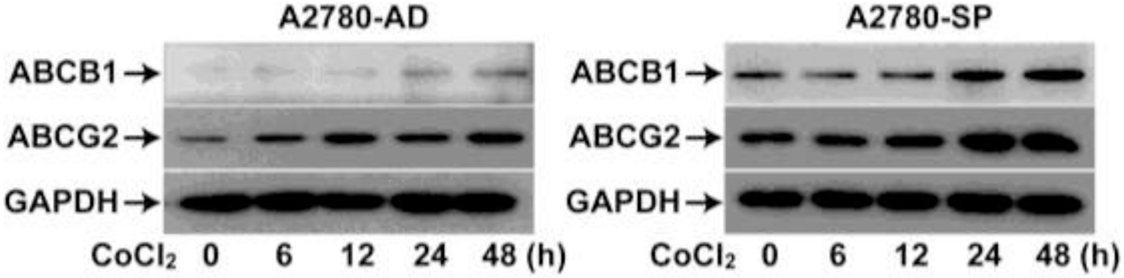

Supplementary Figure S5: $CoCl_2$ treatment increases the expression of ABC trasnporters. Western blot analysis results of adherent cells (AD) and spheres (SP) of A2780 cells after incubation with $CoCl_2$ (100 µM) for indicated hours are shown.

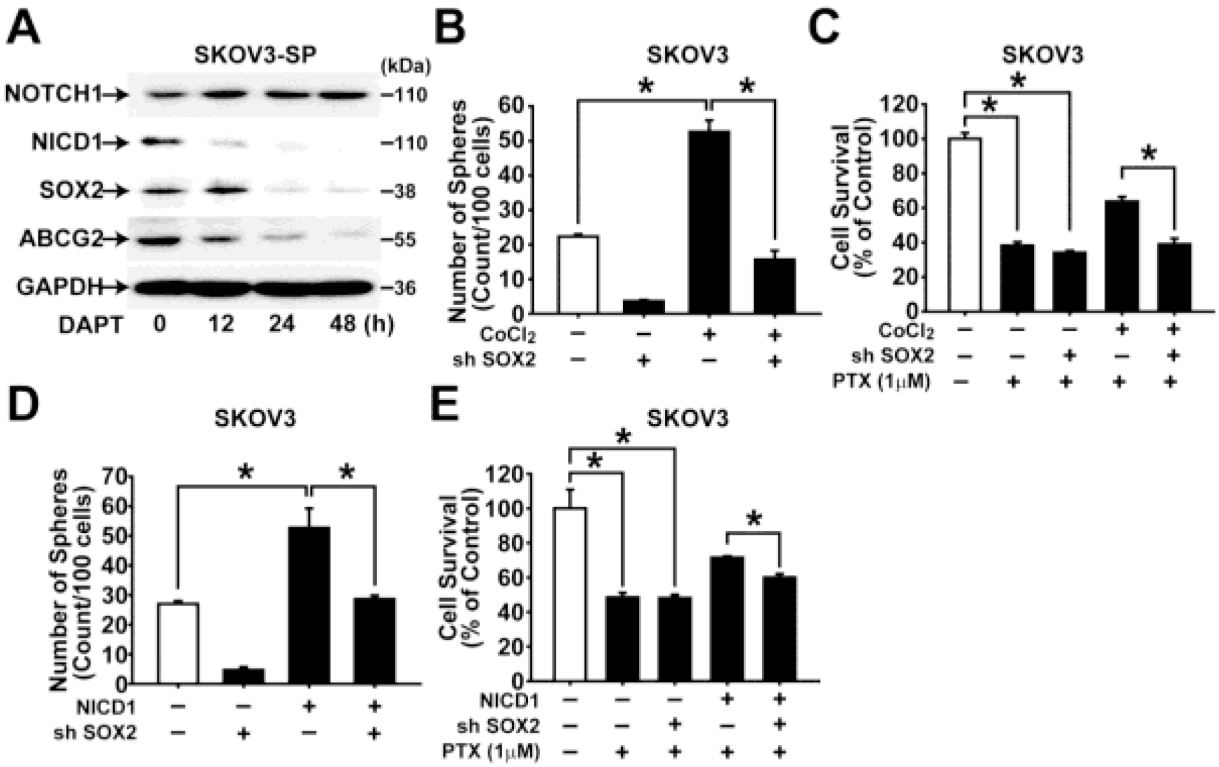

Supplementary Figure S6: NOTCH1 and SOX2 are important for maintaining CSC properties in SKOV3 ovarian cancer cells. **A.** Western blot analysis results of SKOV3 sphere cells after incubation with DAPT (20 μM) for 0, 12, 24, or 48 h are shown with indicated probing antibodies. **B.** Numbers ofspheres generated from adherent SKOV3 cells with or without $CoCl_2$ (100 μM) treatment in combination with SOX2 knockdown are shown. The numbers of spheres were counted on day 10 after treatment. Data indicate mean ± SD (n=3). *, P<0.05. **C.** Viability of adherent SKOV3 cells with or without paclitaxel (1 μM) treatment was determined by MTT assay. Paclitaxel was treated for two days in combination with $CoCl_2$ (100 μM) and/or SOX2 knockdown. The percentage of viable cells is shown after normalization to no treatment control. Data indicate mean ± SD (n=3). *, P<0.05. **D.** Numbers ofspheres generated from adherent SKOV3 cells with NICD1 overexpression in combination with SOX2 knockdown are shown. The numbers of spheres were counted after 10 days. Data indicate mean ± SD (n=3). *, P<0.05. **E.** Viability of adherent SKOV3 cells with or without paclitaxel (1 μM) treatment was determined by MTT assay. Paclitaxel was treated for two days in combination with NICD1 overexpression and SOX2 knockdown. The percentage of viable cells is shown after normalization to no treatment control. Data indicate mean ± SD (n=3). *, P<0.05.

Homo sapiens SRY (sex determining region Y)-box 2 (SOX2) 1545

```
                                                        TAG CGACAACAAG AGAAacaaaa
3541 ctacctattt GTAACGGACG TGCTGCCATT gccctccgca TTGAGCGCCT ACCTATTgaa
3601 atctttacgt cgggacaatg ggagagcggc taaaattacc ctcttgggtc ctgggcgggc
3661 aagattcctg agcccctacc cccgccccca tctcatcctc ctctaacccg ggccttgctg
3721 ggctccccct tccccagtcc cggccgcctt ctcccagtgt gcgctgcctg cacctgtgcc
3781 tggagagcat cgaccccgcc tcccaggcct tgagcccctt tgcggcgcag ccccagcctt
3841 gcgcggcctg ggctttgcgg ccaccacaat ggaaatctac ggggaaaatg ccagggctgg
3901 ttctgctgga gtccTGGGaa ctc tgcgTGG Gaggga gttt gtgactgcgg cccaaaagcc
                  CSL-3          CSL-2
3961 acctccatac agtgccgTGG Gatgccagga agttgaaatc accctccccc atcgcctgca
                 CSL-1
4021 cttttgagcg cccttccgtc tgtgtctttc cccagccccc atttgaaagc cgcacgaccg
4081 aaacccttct tacggggagg catgggatgg gaatggggag tgggggcaga cagtagaagc
4141 atcccctttg CTACGGTTGA ATGAAGACAG TCTAGTGgga gatgtggctg gggctaagag
4201 gaagagctgc agtttcctgg gccaaagagc tgagttggac agggagatgg cagcttacca
4261 aggcctgctg gttctcagct ctagagtctg ccttatggtc cgagcaggat ttatttttaa
4321 gaacagagca agttacgtgg aagcaaggaa ggttttgagg acagaggttt gggtctccta
4381 acttctagtc gggactgtga gaAGGGCGTG AGAgagtgtt ggcacctgta aggtaagaga
                     HIF-1 ancillary sequence-1
4441 ggagagcgga agagcgcagt acgggagcgg caccagaggg gctggagttg gggggagtg
4501 ctgtggatga gcgggagaac aatgacacac caactcctgc actggctgtt tccagaaata
4561 cgagttggac agccgccctg agccacccac tgtgccctgc cccacccccg caccttagct
4621 gcttcccgcg tcccatcctc atttaagtac cctgcaccaa aaagtaaatc aatattaagt
4681 ttaaagaaaa aaaaacccac gtagtcttag tgctgtttac ccacttcctt CGAAAAGGCG
4741 TGTGGtgtga cctgttgctg cgagagggga tacaaaggtt tctcagtggc tggcaggctg
  HIF-1 ancillary sequence-2
4801 gctctgggag cctcctcccc ctcctcgcct gccccctcct cccccggcct cccccgcgcg
4861 gccggcggcg cgggaggccc cgCCCCCTTT CATGCAAAAC CCggcagcga ggctgggctc
4921 gagtggagga gccgccgcgc gctgattgGT CGCTAGAAAC CCATTTATTC CCTgacagcc
4981 cccgtcacat ggatggttgt ctattaactt gttcaaaaaa gtatcaggag ttgtcaaggc
5041 agagaagaga gtgtttgcaa aaggggaaa gtagtttgct gcctctttaa gactaggact
5101 gagagaaaga agaggagaga gaaagaaagg gagagaagtt tgagccccag gcttaagcct
```

Supplementary Figure S7: SOX2 promoter analysis. HIF-1 binding elements and CSL binding elements in 1.5Kb 5' upstream region of SOX2 promoter are shown.

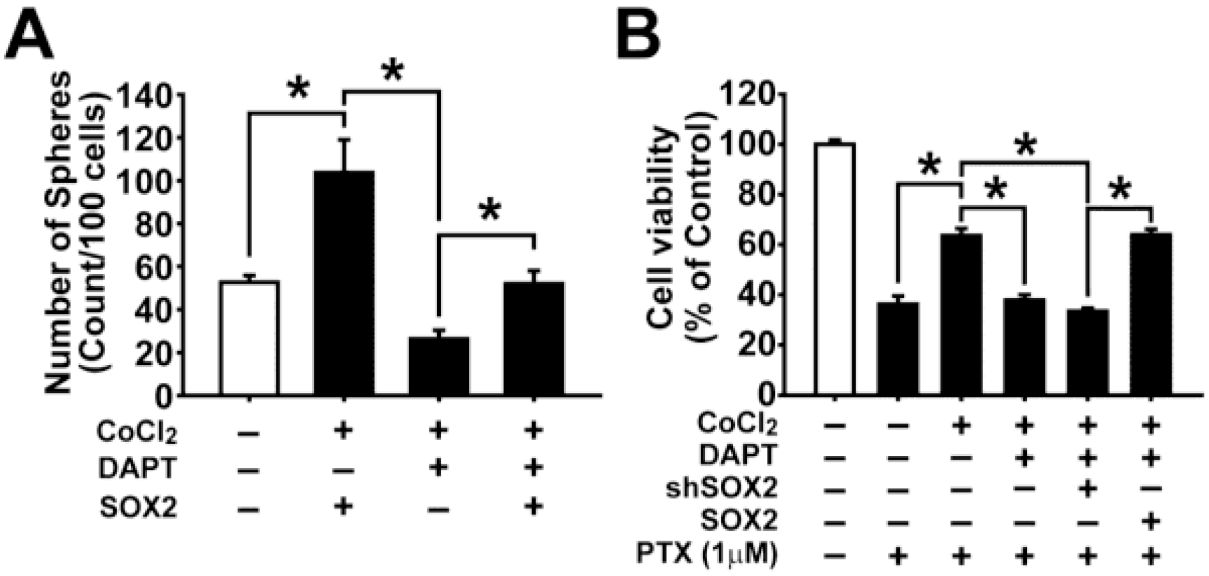

Supplementary Figure S8: Hypooxia-NOTCH-SOX2 signaling axis is important for maintaining CSC characteristics in ovarian cancer cells. A. Sphere numbers generated from A2780 cells with combination treatments of $CoCl_2$ (100 μM), DAPT (10 μM), and SOX2 overexpression are shown. Data indicate mean ± SD (n=3). *, P<0.05. **B.** Viability of A2780 cells was determined by MTT assay after combination treatments with $CoCl_2$ (100 μM), DAPT (10 μM), paclitaxel (1 μM) along with SOX2 knockdown or SOX2 overexpression. The percentage of viable cells is shown after normalization to no treatment control. Data indicate mean ± SD (n=3). *, P<0.05.
